# Comparing second cancer risk for multiple radiotherapy modalities in survivors of hodgkin lymphoma

**DOI:** 10.1259/bjr.20200354

**Published:** 2021-04-09

**Authors:** Claire Timlin, James Loken, Jon Kruse, Robert Miller, Uwe Schneider

**Affiliations:** 1 Particle Therapy Cancer Research Institute, University of Oxford, Oxford, UK; 2 Mayo Clinic, Rochester, MN, USA; 3 Department of Physics, University of Zurich and Radiotherapy Hirslanden, Zurich, Switzerland

## Abstract

**Objectives:**

To assess if excess absolute risk (EAR) of radiation-induced solid cancer can be used to rank radiotherapy plans for treatment of Hodgkin lymphoma (HL) in a statistically significant way.

**Methods:**

EAR models, calibrated with data from the Life Span Study and HL survivors, have been incorporated into a voxelised risk-calculation software, which is used to compare four treatment modalities planned for five virtual HL patients. Organ-specific parameters are generated repeatedly in a Monte Carlo fashion to model their uncertainties. This in turn enables a quantitative estimation of the EAR uncertainties.

**Results:**

Parameter-driven uncertainties on total EAR are around 13%, decreasing to around 2–5% for relative EAR comparisons. Total EAR estimations indicate that intensity modulated proton therapy decreases the average risk by 40% compared to the intensity modulated radiation therapy plan, 28% compared to the volumetric modulated arc therapy plan whereas the three-dimensional conformal radiation therapy plan is equivalent within the uncertainty.

**Conclusion:**

Relative EAR is a useful metric for distinguishing between radiotherapy plans in terms of second cancer risk.

**Advances in knowledge:**

Relative EAR is not dominated by model or parameter uncertainties and can be used to guide the choice of radiotherapy for HL patients.

## Introduction

The life span study (LSS) of atomic bomb survivors has shown that radiation leads to an enhancement in the risk of cancer compared with unirradiated groups.^1^ Whilst there is a debate about the functional form of the dose response at higher doses,^2^ at low doses there is no evidence for a threshold, below which the risk can be considered negligible. An absolute excess risk is also evident in clinical cohorts treated for Hodgkin lymphoma (HL) with radiotherapy (RT),^3,4^ where an elevated risk is observed as much as 30 years post-treatment.

For the cohort studied in Aleman et al,^4^ 95% of treatment-related deaths from second cancer at 30 years occurred after the first 10 years follow-up. This demonstrates the importance of long-term follow-up to assess second-cancer risk. This slow appearance of second cancers makes quantifying risk challenging, as it is necessary to use risk curves fitted to historic data to predict risk from modern radiotherapy plans.

For patients who have received RT and/or chemotherapy for HL, second cancers are a major cause of treatment-related morbidity and ongoing treatment-related costs. It has been shown that in some cases, treatment-related morbidity due to second solid-cancers is more common than cardiovascular disease^4,5^ and that third and fourth cancers also occur at a rate above control groups.^6^ The 10 year survival for HL sufferers is now greater than 80% in England and Wales, and since the disease affects both young and old, it is entirely possible for a young patient to survive long enough to experience multiple other malignancies.^6^ It is therefore of utmost importance to discover if these risks can be reduced, and by how much, with careful design of treatment protocols to minimise treatment-induced risk.

Modern radiotherapy modalities can be planned such that they are equivalent with regards to cure probability and severe acute and late toxicity to normal tissues. Techniques such as intensity modulated X-ray therapy (IMRT) and volumetric modulated arc therapy (VMAT) produce high-dose regions which closely conform to the target volume. This is generally achieved by substantially increasing the volume of healthy tissue exposed to low doses, which could lead to an increase in treatment-induced second cancers. Proton treatments can be planned which similarly conform to the treatment volume but with a reduction in low-intermediate dose outside target when compared with VMAT and IMRT. It is important to understand the impact of these treatment options in terms of radiation-induced second cancer.

This paper details a methodology to estimate excess absolute risk (EAR) of second solid cancer induced via radiotherapy. The models used are calibrated with data from the LSS and HL survivors.^7^ Risk is estimated by a software tool, using the three-dimensional prescribed dose distribution for each modality along with patient-specific anatomy. The modalities are then compared for five virtual HL patients. An earlier version of this software tool is described in more detail in.^8^ For this analysis, it has been completely refactored to include organ-specific, clinical data-driven dose–response curves. Five virtual patients, with plans designed to treat five different stages of HL, are analysed. As the confidence in estimating second cancer risk is limited by uncertainty in the input parameters to the dose–response curves, the effect of these uncertainties on the calculated EAR is also quantified. This is achieved by varying the model parameters within tissue-specific confidence intervals. The focus is on assessing if the RT plans can be ranked in terms of EAR, despite the underlying uncertainties in the EAR models.

## Methods and materials

### The FULL excess absolute risk model

The excess absolute risk of second cancer induction was estimated as a function of dose, *D*, using the mechanistic models of carcinoma and sarcoma induction developed by Schneider.^7,9^ This is achieved through the use of the FULL model, which estimates risk equivalent dose (RED) for organs allowing for repopulation/repair-ability of tissue. RED was fitted for all organs in this study to epidemiological second cancer data in the high-dose range. The model is detailed fully in Schneider^9^ and summarised in this section.

The FULL model for RED is expressed as:

(1) $$RED(D)=\frac{e^{-\alpha^{\prime }D}}{\alpha^{\prime }R}\left(1-2R+R^{2}e^{\alpha^{\prime }D}-\;(1-R{)}^{2}e^{-\frac{\alpha^{\prime }R}{1-R}D}\right)$$

(2) $$RED(D)=\frac{e^{-\alpha^{\prime }D}}{\alpha^{\prime }R}\left(1-2R+R^{2}e^{\alpha^{\prime }D}-\;(1-R{)}^{2}e^{-\frac{\alpha^{\prime }R}{1-R}D}-\alpha^{\prime }RD\right)$$

for carcinoma and sarcoma induction, respectively. The parameter *R* characterises the repopulation/repair-ability of the tissue between two dose fractions. It is 0 if no repopulation/repair and 1 if full repopulation/repair occurs.^9^ *α*’ characterises the cell killing and is determined according to the linear-quadratic model:

(3) $$\alpha^{\prime }=\alpha +\beta d$$

where *α* and *β* are the usual radiosensitivity parameters and *d* is the dose delivered per fraction. The values for *α* were taken from Schneider et al,^7^ where equations 1 and 2 were fitted to epidemiological data using a constant *α* over *β* ratio of 3. It should be noted that *R*, *α* and *β_EAR_* are tissue-specific model parameters.

There are separate models for carcinoma and sarcoma induction risk. This is because the excess risk of sarcomas observed from the study of the A-bomb survivors is an order of magnitude smaller than for carcinomas. However, data from radiotherapy patients indicate that sarcoma induction at high dose is at a comparable magnitude to carcinoma induction. Therefore, it is not appropriate to assume a pure linear-exponential dose–response relationship for sarcoma induction. A sarcoma induction model was used^7,9^ which accounts for cell killing and fractionation effects, and is based on the assumption that stem cells remain quiescent until external stimuli, like ionising radiation, trigger re-entry into the cell cycle.

Two data sets were used to connect these mechanistic models to excess absolute risk: the atomic bomb survivor data at low doses,^10^ and second cancer risk data for a HL cohort at doses relevant for radiotherapy.^3^ Tissue-specific model parameters were determined by fitting these data to the mechanistic models,^7^ using the expression

(4) $$EAR=\beta_{EAR}\times RED(D)\times \mu (t_{exp},t_{att})$$

where *β_EAR_* is the gradient of the dose–response curve at low doses (where the linear-no-threshold model is valid) and is taken directly from the atomic bomb survivor data analysis.^10^

The function *µ* modifies the risk according to age at exposure, *t*_exp_, and their attained age,*t*_att_

(5) $$\mu (t_{exp},t_{att})=e^{\left(\gamma_{exp}(t_{exp}-30)+\gamma_{att}ln(t_{att}/70)\right)}$$

where the parameters *γ*_exp_ and *γ*_att_ are again taken directly from the atomic bomb survivor data analysis.^10^ For the results presented in this paper, the age at exposure is assumed to be 30 years and the attained age is assumed to be 70 years, and therefore *µ* is 1. The best-fit parameter values and their confidence intervals are shown for each tissue type in (Tables 1–3).

**Table 1.** Risk equivalent dose input parameters for FULL model of carcinoma induction

Site	α (*Gy*^*−1*^*)*		*R*		Comments
	mean	σ	mean	σ	
Breast	0.0440	0.0950	0.1500	0.0700	
Oesophagus	0.4600	0.0750	0.4600	0.2250	Estimated using stomach values
Heart	–	–	–	–	Negligible risk of second solid cancer
Liver	0.3230	0.9050	0.2900	0.0950	
Lungs	0.0420	0.0550	0.8300	0.0750	
Pharynx	0.0430	0.0095	0.9700	0.0700	
Spinal cord	0.0180	0.0085	0.9300	0.2550	
Stomach	0.4600	0.0750	0.4600	0.2250	
Thyroid	0.0318	0.0075	0.0000	0.0000	
Vessels	–	–	–	–	Negligible risk of second solid cancer.

The mean values are from Table 4 in Schneider et al^7^. The standard deviations (*σ*) on *α* and *R* have been obtained from the code detailed in Schneider et al.^7^ The thyroid parameter values are from Tomozeiu^13^ .

**Table 2.** Risk equivalent dose input parameters for full model of sarcoma induction, assuming full tissue recovery, taken from Table 5^7^

Site	*β_EAR_* (10*,*000*PY Gy*)^−1^		*α* (*Gy*^−1^)	
	mean	*σ*	mean	*σ*
Bone	0.1	4.3 × 10^−4^	0.078	4.3 × 10^−4^
Soft tissue	0.35	2.03 × 10^−4^	0.093	2.03 × 10^−4^

PY, per person per year.

**Table 3.** *β_EAR_* are taken from Table 1 in Schneider et al,^7^ the data for which originated from Preston et al^10^

Site	β_EAR_ (10*,*000*PY Gy*)^−1^		
	Mean	−σ	+σ
Breast	8.2	1.05	1.4
Oesophagus	3.2	1.1	1.45
Liver	2.4	1.2	0.8
Lungs	8.0	1.25	1.5
Pharynx	0.73	0.215	0.435
Spinal cord	0.04	0.015	0.019
Stomach	5.2	0.9	1.25
Thyroid	0.4	0.1	0.2

CNS, central nervous system; PY, per person per year.

95% CI have been used to calculate standard deviation (*σ*) by assuming a normal distribution. The spinal cord *β* value has been taken from “Brain and CNS” and rescaled to the partial volume of the spinal cord with respect to brain and spinal cord.

In the limit of low dose, both the carcinoma and sarcoma models reproduce the linear-no-threshold relation observed in the LSS. Whereas at high dose, the behaviour depends upon the tissue-specific model parameters.

### Hodgkin lymphoma radiotherapy plans

Five different stages of HL, hence five virtual patients, have been investigated in this analysis with the clinical case histories summarised below:

HL, ABVD (Adriamycin, Bleomycin, Vinblastine, Dacarbazine) chemotherapy, five PTVs (planning target volume) encompassing:

- Virtual Patient 1: Stage IA (involvement of upper mediastinum)
- Virtual Patient 2: Stage IIA (involvement of upper mediastinum and lower neck and bilateral supraclavicular nodes)
- Virtual Patient 3: Stage IIA (involvement of upper and lower mediastinum and lower neck and bilateral supraclavicular nodes)
- Virtual Patient 4: Stage IIA (involvement upper and lower mediastinum, left axilla, and lower neck and bilateral supraclavicular nodes)
- Virtual Patient 5: Stage IIA (involvement upper and lower mediastinum, left axilla, pericardial nodes, and lower neck and bilateral supraclavicular nodes)

Radiotherapy treatment plans were constructed on the same patient anatomy for each of the five disease stages. The modalities planned include spot scanned protons (IMPT) and three photon plans: 3D conformal radiotherapy (3DCRT), IMRT using static treatment fields and VMAT for which intensity modulation would be administered during the gantry rotation. The prescription dose for all plans was 30 Gy in 15 fractions to the PTV, with the PTV encompassing larger volumes from patient 1 to 5. The planning priorities were the same for all modalities and are detailed in (Table 4). Treatment plans were generated using Varian Eclipse software.

**Table 4.** Planning priorities for HL RT plans. VX <Y% means that Y % of the structure volume should receive less than X dose [Gy]

**Priority 1 lung**
V5 <40%
V10 <30%
V13 <25%
V20 <20%
Mean lung dose <14 Gy
**Priority 2 PTV target dose: 30.0 Gy in 15 fractions**
>95% vol of the PTV to receive the prescribed dose.
<20% vol of the PTV to receive >105% of the prescribed dose.
<1% vol of the PTV to get <93% of the prescribed dose.
<1% vol or <1 cc of unspecified tissue outside of the PTV to receive >105% of the prescribed dose.
**Priority 3**
Spinal cord +5 mm: 95% vol <45 Gy; 100% vol < 50 Gy
Heart: V30 <50% and mean heart dose <20 Gy

HL, Hodgkin lymphoma; PTV, planning target volume; RT, radiation therapy.

It is important to note that the results presented in this paper are dependent on the specific dose plans. The estimated risks will depend on beam directions, and the number of beams used, so should be regarded as specific to these plans rather than generalised to the modality. It is hoped that methods of voxelised relative risk estimation, like ours, could be used in the future to optimise plans in terms of radiotherapy-induced EAR.

Despite each modality having the same set of planning priorities, the planned DICOM-RT dose cubes can differ considerably. Supplementary Tables 1–4 summarise the organ and target doses for virtual patients 1 and 5. Throughout this paper, these have been used as examples as they are the extreme stages of HL, Stage IA (involvement of upper mediastinum) and Stage IIA (involvement upper and lower mediastinum, left axilla, pericardial nodes, and lower neck and bilateral supraclavicular nodes) respectively.

Supplementary Table 1.

Supplementary Table 2.

Supplementary Table 3.

Supplementary Table 4.

### EAR model implementation in HL radiotherapy plans

#### Voxel-by-voxel RED calculation

Radiotherapy plans can be divided into voxels the size of the CT scan resolution (0.1055 × 0.1055 × 0.2500 cm^3^). A software framework was developed which calculates a per-voxel risk of second cancer for a given treatment plan.^8^ It is written using MATLAB (2013b, MathWorks)^11^ and employs functionality from CERR (computational environment for radiation research).^12^ The framework is designed to operate on plans in the DICOM-RT format importing planning CT, structure sets and dose cubes.

**Figure 1.**
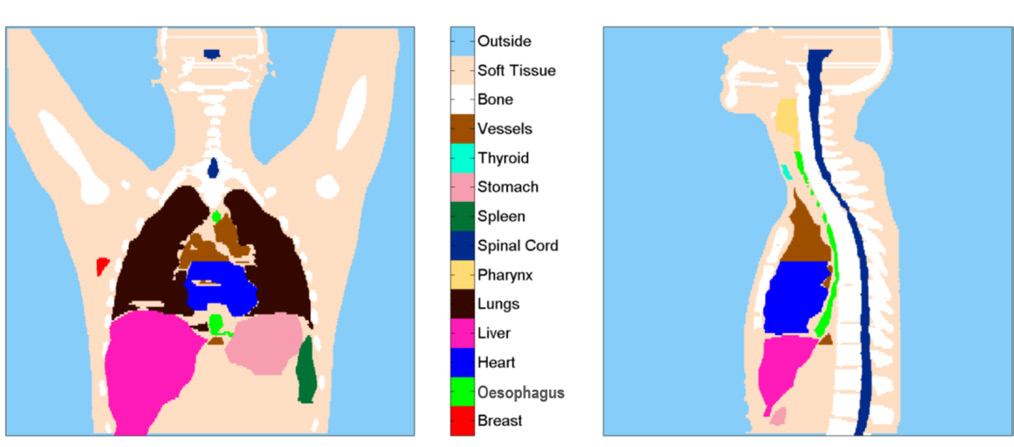
Coronal slice 276/512 and sagittal slice 260/512 of the single valued, normal tissue, structure matrix for the anatomy on which all the HL treatments were planned. Pharynx includes hypopharynx and oropharynx. HL, Hodgkin lymphoma.

Firstly, structures and doses are imported and necessary data extracted. Where a voxel has more than one tissue flag, the most specific one is retained, such that all voxels within the patient are assigned to precisely one structure. Figure 1 shows a slice through the resulting, single valued, structure matrix for the anatomy on which all the HL treatments were planned. The structure matrix is used to assign model parameters, appropriate to that organ, to each voxel within that organ via a look-up table. A corresponding dose map is then calculated on the same voxel grid by linear interpolation of the DICOM-RT dose description.

RED is calculated for each voxel using the FULL excess absolute risk model described previously and fully detailed in Schneider et al.^7^ For carcinoma induction, *RED* is calculated as in Equation 1, using mean parameters in Table 1. For bone and soft tissue parameters, Equation 2 for sarcoma induction has been employed, with parameters given in Table 2.

When applying these RED models to the proton plan, the biological effectiveness, relative to photons, must be considered. A relative biological effectiveness (RBE) of 1.1 has been assumed for every voxel in the treatment volume and it is the RBE weighted dose that is D in the RED equations.

### Organ-specific EAR calculation

The EAR of each organ is calculated by summing the voxel RED in that organ and multiplying by the relevant *β_EAR_* value:

(6) $$EAR_{org}=\mu \frac{\beta_{EAR}}{N_{org}}\sum_{i}‍RED_{i}$$

*N*_org_ is the total number of voxels in the organ. For this analysis, the patient is assumed to be 30 years old at time of treatment and to attain and age of 70, hence *µ* is equivalent to 1 by definition.

The *β_EAR_*, *α* and *R* values all have associated uncertainties which contribute to an uncertainty on the calculated EAR for each organ. The organ-specific uncertainties on *β_EAR_* are detailed in Schneider et al^7^ and are listed in Table 3. The uncertainties on *α* and *R*, detailed in Table 1, were obtained by independent fits to the observed EAR variations of Dores et al.^3^ Note that the EAR models all predict zero risk at zero dose, so non-target organs are not considered in this analysis.

Where the confidence intervals (CIs) for the organ-specific parameters are asymmetric, the highest standard deviation was applied to both the positive and negative standard deviations. This is a worst-case scenario that will lead to an overestimate of the errors. All input parameters were assumed to be normally distributed.

To quantify the uncertainty introduced in EAR from these input uncertainties, a job management system was developed which allows the calculation of organ-specific EAR to be performed many times for different sets of inputs. It uses a Monte Carlo approach, randomly sampling a normal distribution, around each parameter’s average value, hence three normal distributions are used for *β_EAR_*, *α* and *R* for each organ. The software produces an EAR distribution for each organ, for each modality, for each virtual patient, to which a normal distribution is fitted.

### Total solid cancer EAR estimation

The total EAR of radiation-induced second solid cancer to a virtual patient is the sum of risk over all irradiated organs. There are 260,000 runs computed in total: 1000 for each of the 5 virtual patients, 4 modalities and 13 organs. This produces an EAR distribution for each plan and virtual patient, to which a normal distribution can be fitted to attain the mean and standard deviation.

### Relative EAR calculation

Relative EAR can be defined for a specific organ (RelEAR_org_). It is defined as the ratio of EAR for two given treatment plans that are planned on the same virtual patient. It should be emphasised that relative in this sense means the risk of plan A relative to plan B. Because *β* is the same for both plans when a specific organ is considered RelEAR_org_ becomes a simple ratio of RED:

(7) $$RelEAR_{org}=\frac{\sum_{i=1}^{N}‍RED(D_{i}{)}^{Plan1}}{\sum_{i=1}^{N}‍RED(D_{i}{)}^{Plan2}}$$

where *i* denotes a voxel in a given organ and N is the total number of voxels in that organ.

Relative EAR for second solid cancer can be defined for a virtual patient (RelEAR_tot_) but the formula cannot be reduced to a simple ratio of REDs. It is defined as the ratio of the sum of EAR, over all organs, for two given treatment plans:

(8) $$RelEAR_{tot}=\frac{\sum_{j=1}^{n}‍EAR_{j}^{Plan1}}{\sum_{j=1}^{n}‍EAR_{j}^{Plan2}}$$

where *j* denotes a specific organ and *n* is the total number of organs in the treatment volume. The feasibility of using this metric to rank treatment plans in terms of second cancer risk is the focus of this paper.

## Results

**Figure 2.**
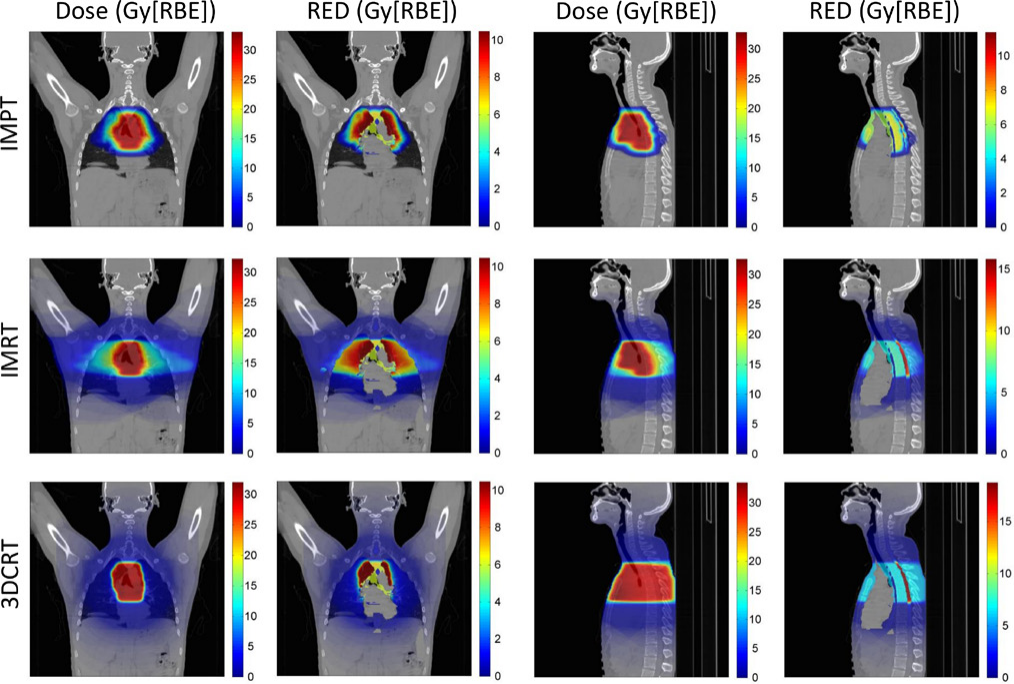
Virtual patient 1: 2D coronal and sagittal voxel maps of dose and estimated RED for IMPT, IMRT and 3DCRT treatment plans. Coronal slice 276/512 and sagittal slice 260/512 shown. 2D, two-dimensional; 3DCRT, three-dimensional conformal radiotherapy; IMPT, intensity modulated proton therapy; IMRT, intensity modulated radiotherapy; RBE, relative biological effectiveness; RED, risk equivalent dose.

**Figure 3.**
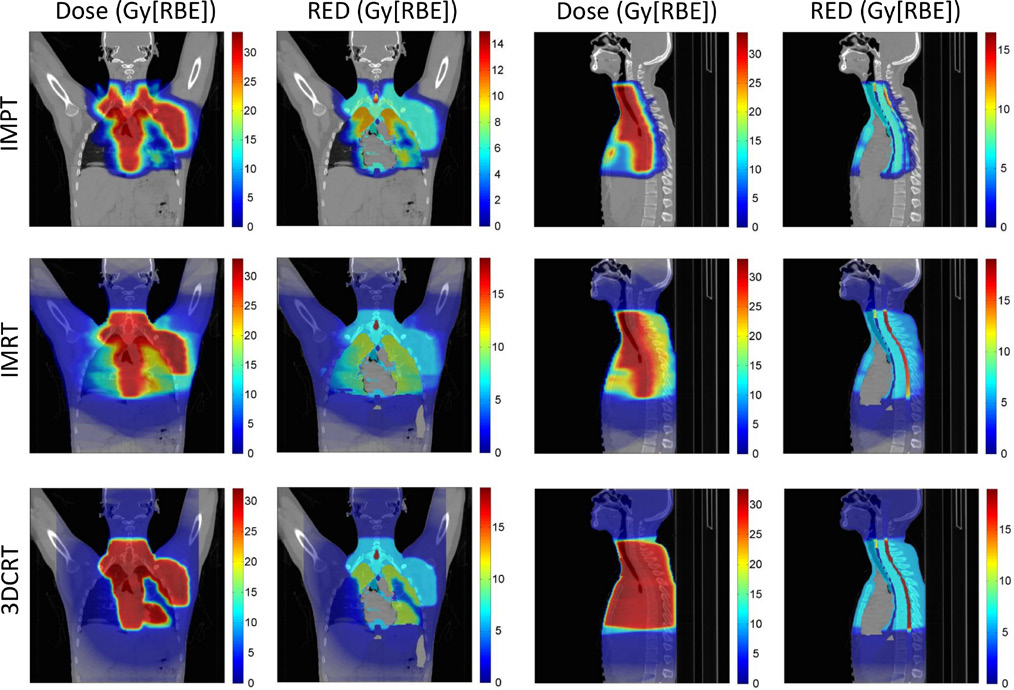
Virtual patient 5: 2D coronal and sagittal voxel maps of dose and estimated RED for IMPT, IMRT and 3DCRT treatment plans. Coronal slice 276/512 and sagittal slice 260/512 shown. 2D, two-dimensional; 3DCRT, three-dimensional conformal radiotherapy; IMPT, intensity modulated proton therapy; IMRT, intensity modulated radiotherapy; RBE, relative biological effectiveness; RED, risk equivalent dose

Voxel-by-voxel RED. Figures 2 and 3 show 2D voxel maps of dose and RED for IMPT, IMRT and 3DCRT treatment plans for virtual patients 1 and 5 respectively. For very low doses, the dose and RED values are similar but deviate at higher doses, as is expected with the FULL, non-linear model. RED is less than (or equal to) dose for all voxels. Organ-specific sensitivities can be observed, *e.g.* the same dose produces a higher RED in the spinal cord than the surrounding tissues. There is no risk of second solid cancer in the heart and vessels as both are assumed to have a negligible risk of radiation-induced second solid cancers. Due to the reduction in integral dose possible with the proton plan, with respect to the photon plans, there are a larger number of voxels that have zero dose, and hence zero RED, and therefore do not contribute to the patient’s risk of treatment-induced second cancer. Virtual patient 5 has higher stage HL than virtual patient 1, and hence the target volume is larger for all plans.

### Organ-specific EAR

**Figure 4.**
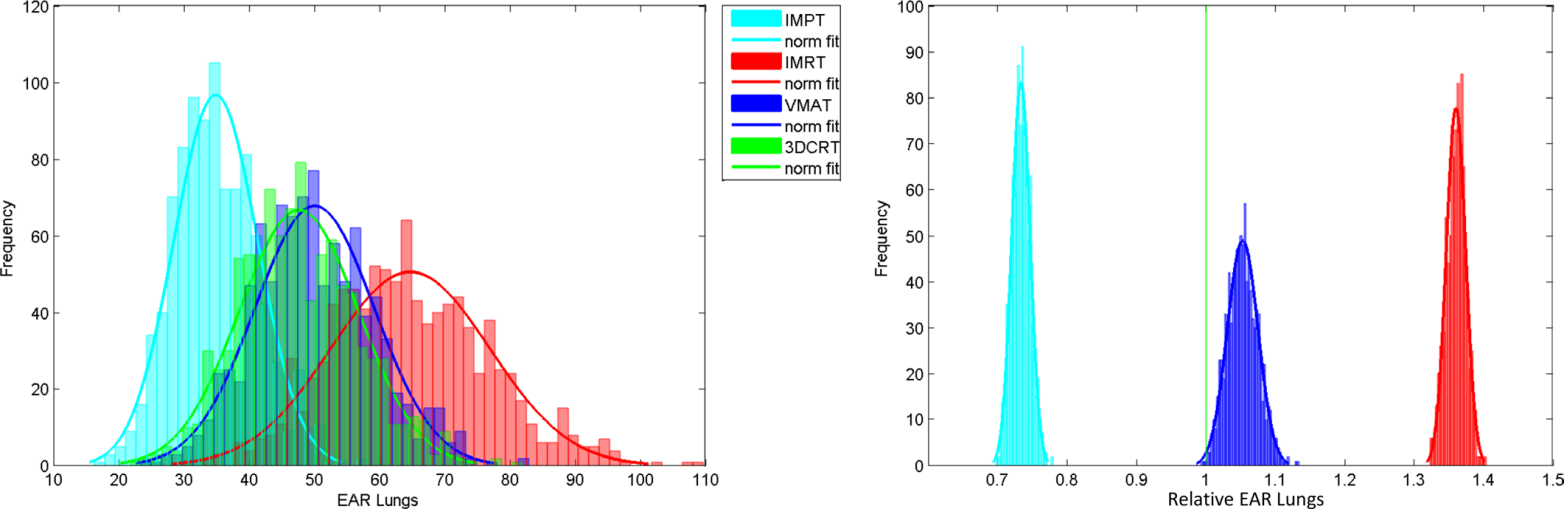
Left: Lung EAR distributions for virtual patient five for IMPT, IMRT, 3DCRT and VMAT. Right: Relative lung EAR distributions for virtual patient 5 for IMPT, IMRT and VMAT normalised to the 3DCRT plan. Relative EAR for 3DCRT is exactly 1 and standard deviation is exactly 0. 3DCRT, three-dimensional conformal radiotherapy; EAR, excess absolute risk; IMPT, intensity modulated proton therapy; IMRT, intensity modulated radiotherapy; VMAT, volumetric modulated arc therapy.

The organ-specific EAR values were calculated by summing *RED_i_* for all voxels in that organ and weighting by *β_EAR_* as shown in Equation 6. Due to the distributions generated for the input parameters, the resulting EAR is also a distribution. Figure 4 (left) shows EAR distributions for the lungs for all treatment modalities for virtual patient 5. The model was run 4000 times for each virtual patient (1000 for each modality) using the same 1000, Monte Carlo generated, input parameter sets. The uncertainty on EAR is appreciable which makes it difficult to decide which plan would be best, solely in terms of lung EAR, for this patient.

If it is assumed that whatever a patient’s unique set of inputs are, they are the same whether the risk is calculated for IMPT, IMRT, 3DCRT or VMAT, then it is possible to calculate the ratio of organ risk with respect to another plan. As an example, the distributions for lung relative EAR, normalised to the 3DCRT plan, are shown in Figure 4 (right). There is still a width to the relative EAR distributions because the dose matrices are different between the modalities and therefore the uncertainties do not completely cancel. However, this is greatly reduced when compared to the absolute EAR. The 3DCRT plan has been used in the denominator of the ratio, hence for this modality, relative EAR is exactly 1 and the uncertainty is 0. The figures show that for the lungs in virtual patient 5, it is possible to resolve the plans, and therefore rank them in terms of risk, using relative EAR.

Tables 5 and 6 display estimates of absolute and relative EAR means and coefficient of variation (CV, ratio of the standard deviation to the mean) for all organs and modalities, for virtual patients 1 and 5 respectively. For virtual patients 1 and 5, the dominant organs by far, in terms of EAR, are lungs and breast. It is these organs which drive the total EAR, and therefore sparing dose in these organs can dramatically affect risk. It should be noted that 3DCRT can produce lower EAR values in these organs than IMPT, as the 3DCRT plan has high dose throughout the patient in the target region with a steep fall off in dose laterally. It should be noted that 3DCRT has other disadvantages, the three other modalities conform better to the isodoses of the planning target volume and therefore better protect the organs at risk.

**Table 5.** Absolute and relative EAR (Equations 6 and 7) for all organs and modalities for virtual patient 1

Structure	EAR ($10,000PY^{-1}$)								EAR Relative to 3DCRT							
	IMPT		3DCRT		IMRT		VMAT		IMPT		3DCRT		IMRT		VMAT	
	Mean	CV	Mean	CV	Mean	CV	Mean	CV	Mean	CV	Mean	CV	Mean	CV	Mean	CV
Breast	12.4	19%	4	16%	16.2	17%	15.4	16%	3.05	5%	1	0%	4	2%	3.81	1%
Oesophagus	0.9	59%	1.7	45%	1.6	45%	1.7	45%	0.49	22%	1	0%	0.94	0%	0.99	1%
Heart	–	–	–	–	–	–	–	–	–	–	–	–	–	–	–	–
Liver	0	0%	0.2	50%	0.1	50%	0.2	45%	0	0	1	0%	0.52	0%	0.96	0%
Lungs	20.6	19%	17.4	19%	32.5	19%	31.2	18%	1.18	1%	1	0%	1.87	1%	1.79	2%
Pharynx	0	0	0.2	30%	0.1	20%	0.1	50%	0	0%	1	0%	0.34	0%	0.84	0%
Spinal Cord	0	0%	0.1	50%	0.1	30%	0.1	30%	0.24	16%	1	0%	0.68	8%	0.73	8%
Spleen	–	–	–	–	–	–	–	–	–	–	–	–	–	–	–	–
Stomach	0	0%	0.6	22%	0.3	23%	0.5	20%	0	0%	1	0%	0.52	0%	0.8	0%
Thyroid	0	0%	0.2	35%	0.1	35%	0.3	35%	0	0%	1	0%	0.63	0%	1.24	0%
Vessels	–	–	–	–	–	–	–	–	–	–	–	–	–	–	–	–
Bone	0	0%	0.1	0%	0.1	0%	0.1	0%	0.5	0%	1	0%	1.01	0%	0.98	0%
Soft tissue	0	0%	0.1	0%	0.1	0%	0.1	0%	0.56	0%	1	0%	1.49	0%	1.29	0%

CV, coefficient of variation; 3DCRT, three-dimensional conformal radiotherapy ; EAR, excess absolute risk; IMPT, intensity modulated proton therapy; IMRT, intensity modulated radiotherapy; PY, per person per year.

“–” indicates this organ is excluded either because there is considered no risk of second solid cancer (*e.g.* heart) or this structure is out of field.

CV, coefficient of variation, which is the ratio of the standard deviation to the mean.

**Table 6.** Absolute and relative EAR (Equations 6 and 7) for all organs and modalities for virtual patient 5

Structure	EAR ($10,000PY^{-1}$)								EAR Relative to 3DCRT							
	IMPT		3DCRT		IMRT		VMAT		IMPT		3DCRT		IMRT		VMAT	
	Mean	CV	Mean	CV	Mean	CV	Mean	CV	Mean	CV	Mean	CV	Mean	CV	Mean	CV
Breast	27	24%	19.5	24%	39.7	22%	27.6	20%	1.39	2%	1	0%	2.05	3%	1.43	5%
Oesophagus	1.9	60%	2.4	54%	2.4	53%	2.4	54%	0.75	10%	1	0%	1	2%	1.02	2%
Heart	–	–	–	–	–	–	–	–	–	–	–	–	–	–	–	–
Liver	0.1	20%	0.8	41%	0.9	40%	0.9	41%	0.06	13%	1	0%	1.09	4%	1.12	3%
Lungs	34.5	19%	47.1	20%	64	19%	49.5	19%	0.73	2%	1	0%	1.36	1%	1.05	2%
Pharynx	1.2	42%	2	42%	1.8	42%	1.9	43%	0.59	3%	1	0%	0.93	1%	0.99	2%
Spinal cord	0.1	40%	0.3	43%	0.2	55%	0.3	40%	0.32	14%	1	0%	0.85	4%	0.95	0%
Spleen	–	–	–	–	–	–	–	–	–	–	–	–	–	–	–	–
Stomach	0.2	20%	2.8	23%	2.6	23%	2.4	23%	0.06	0%	1	0%	0.93	1%	0.87	2%
Thyroid	2.6	53%	2.8	51%	2.7	51%	2.7	52%	0.94	3%	1	0%	0.98	1%	0.96	1%
Vessels	–	–	–	–	–	–	–	–	–	–	–	–	–	–	–	–
Bone	0.1	0%	0.2	0%	0.2	0%	0.2	0%	0.45	0%	1	0%	1.04	0%	0.95	0%
Soft tissue	0.2	0%	0.5	0%	0.6	0%	0.4	0%	0.48	0%	1	0%	1.2	0%	0.97	0%

CV, coefficient of variation; EAR, excess absolute risk; IMPT, intensity modulated proton therapy; IMRT, intensity modulated radiotherapy; PY, per person per year;VMAT, volumetric modulated arc therapy.

“–” indicates this organ is excluded either because there is considered no risk of second solid cancer (*e.g.* heart) or this structure is out of field.

CV is the ratio of the standard deviation to the mean.

For most organs, in most virtual patients, the estimated EARs for the modern photon plans (IMRT and VMAT) are higher than both 3DCRT and IMPT. Virtual patient 5 has a larger PTV than patient 1 and therefore higher EAR values to all organs for all modalities.

The significance of the separation between the organ relative EAR distributions has been calculated for virtual patients 1 and 5 and is displayed in Supplementary Tables 5 and 6. This metric is useful for quantifying whether relative EAR estimate can be used to significantly rank plans in terms of individual organ risks, or whether the uncertainties make the relative EAR distributions impossible to resolve. For virtual patient 1, all modalities are significantly separated for all organs, with the exception of comparing IMRT with VMAT where breast, lungs and spinal cord are not resolvable. This is because the IMRT and VMAT plans are similar in terms of DICOM dose, so do not differ significantly in terms of EAR for organs in or adjacent to the target volume. For virtual patient 5, most organs have relative EAR that is significantly different for all modalities and is therefore a useful metric for ranking plans in this respect. Some exceptions include breast where IMPT and VMAT distributions overlap, liver where IMRT and VMAT overlap and pharynx where 3DCRT and VMAT overlap.

Supplementary Table 5.

Supplementary Table 6.

### Total EAR of solid cancer

**Figure 5.**
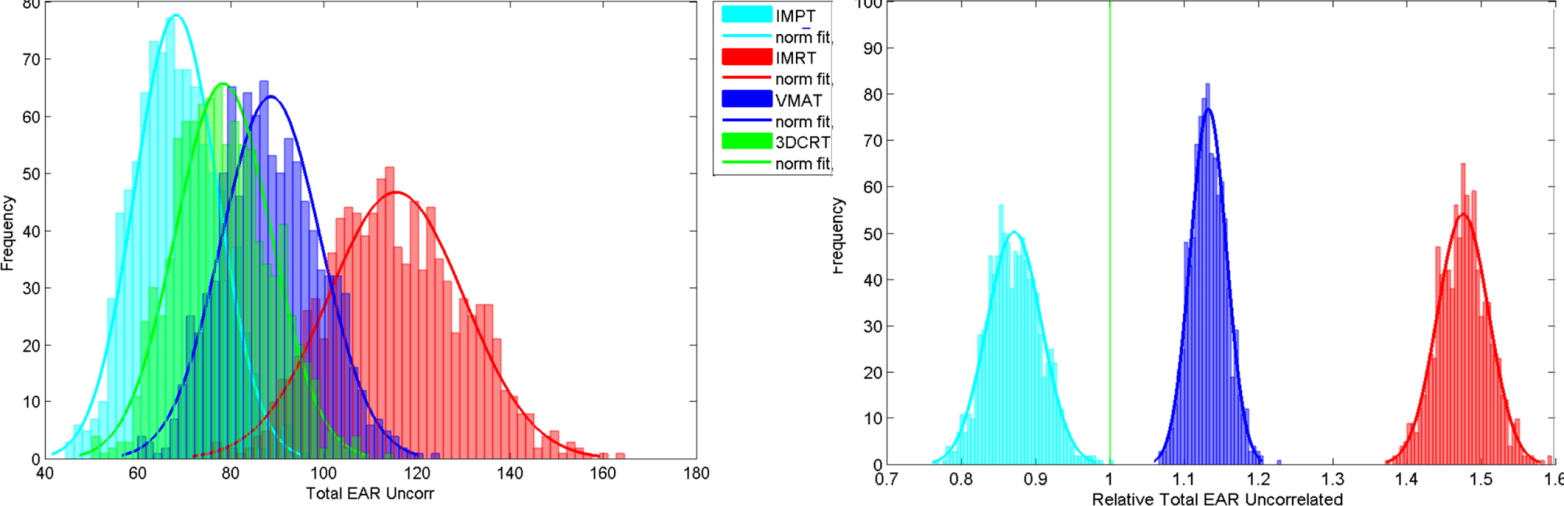
Estimated EAR and relative EAR distributions for virtual patient 5. 3DCRT, three-dimensional conformal radiotherapy; EAR, excess absolute risk; IMPT, intensity modulated proton therapy; IMRT, intensity modulated radiotherapy; VMAT, volumetric modulated arc therapy.

The total EAR of radiation-induced solid second cancer is the sum of risk over all irradiated organs. The total EAR, and relative EAR is shown graphically for virtual patient 5 in Figure 5 and the fit values for all are given in Table 7. The estimated EARs increase, for all modalities, as the PTV region encompasses more tissue. The parameter-driven uncertainties on all absolute EAR values are about 13%, which means most distributions overlap. However, because correlations in the uncertainties are taken into account for relative EAR, the widths of these distributions decrease to between 2 and 5% of mean. Table 7 shows that IMPT or 3DCRT produce the lowest total EAR estimations for all virtual patients with IMRT mostly producing the highest risk values.

**Table 7.** Absolute and relative EAR, summed over all organs for all five virtual patients

	EAR ($10,000PY^{-1}$)								EAR relative to 3DCRT							
	IMPT		3DCRT		IMRT		VMAT		IMPT		3DCRT		IMRT		VMAT	
	Mean	CV	Mean	CV	Mean	CV	Mean	CV	Mean	CV	Mean	CV	Mean	CV	Mean	CV
Virtual Patient 1	34.3	13%	24.9	14%	51.6	13%	50	12%	1.38	5%	1	0%	2.08	4%	2.02	3%
Virtual Patient 2	39.3	12%	36.1	13%	58.1	12%	59.4	12%	1.09	4%	1	0%	1.61	3%	1.65	4%
Virtual Patient 3	50.8	12%	46.9	12%	95.1	12%	76.4	11%	1.08	4%	1	0%	2.03	4%	1.63	3%
Virtual Patient 4	61.1	12%	61.9	12%	104.3	12%	79	11%	0.99	3%	1	0%	1.68	2%	1.28	2%
Virtual Patient 5	68.5	13%	79	13%	116.2	13%	89.2	12%	0.87	4%	1	0%	1.47	2%	1.13	2%

CV, coefficient of variation; 3DCRT, three-dimensional conformal radiotherapy; EAR, excess absolute risk; IMPT, intensity modulated proton therapy; IMRT, intensity modulated radiotherapy; PY, per person per year;VMAT, volumetric modulated arc therapy.

CV is the ratio of the standard deviation to the mean.

Table 8 shows the significance of separation in the relative EAR distributions. For all virtual patients, IMPT is significantly (*p <* 0.001 or three standard deviations) resolvable from both VMAT and IMRT. IMPT and 3DCRT have less than three standard deviations separating the relative EAR distributions for four virtual patients. IMRT and VMAT overlap for virtual patients 1 and 2 but are resolvable for the other three virtual patients.

**Table 8.** Significance of overlap of the relative EAR distributions for all virtual patients and modalities

	IMPT *vs* 3DCRT	IMPT *vs* IMRT	IMPT *vs* VMAT	3DCRT *vs* IMRT	3DCRT *vs* VMAT	IMRT *vs* VMAT
Virtual Patient 1	a	a	a	a	a	0.28
Virtual Patient 2	0.01	a	a	a	a	0.31
Virtual Patient 3	0.02	a	a	a	a	a
Virtual Patient 4	0.37	a	a	a	a	a
Virtual Patient 5	a	a	a	a	a	a

3DCRT, three-dimensional conformal radiotherapy; EAR, excess absolute risk; IMPT, intensity modulated proton therapy; IMRT, intensity modulated radiotherapy; VMAT, volumetric modulated arc therapy.

a Means significant (*<*0.001), *i.e*. more than three standard deviations separating the distributions.

An important question to answer for these dose plans is how many person years can be saved by using a proton plan rather than a modern photon plan, *e.g*. IMRT. From studying IMPT vs. IMRT for all virtual patients, the mean EAR difference is 34.3 per 10,000 person years. In terms of relative risk, the IMRT plans come with a 67% increase in risk relative to IMPT. For IMPT *vs* VMAT for all patients, the mean EAR difference is 20.0 per 10,000 person years. In terms of relative risk, the VMAT plans come with a 39% increase in risk relative to IMPT. IMPT and 3DCRT have been found to not be significantly resolvable for most virtual patients, hence the risk of treatment-induced second cancer should be considered equivalent.

### Linear model comparison

This paper has focused on employing the FULL model described in Schneider et al.^7^ In the interests of completeness, and to assess how sensitive relative EAR is to radical changes in dose–response curve, the total EAR and relative EAR for the following linear model has been estimated using the expression

(9) $$EAR_{LIN}=\beta_{EAR}\cdot D\cdot \mu \left(t_{exp},t_{att}\right)$$

where *β_EAR_* is the gradient of the dose–response curve at low doses and is taken directly from the atomic bomb survivor data analysis.^10^

The total EAR, and relative EAR, for all virtual patients are given in Table 9. The EARs increase, for all modalities, as the target region is expanded from virtual patients 1 to 5. The uncertainties on all absolute EAR values are appreciable (about 14% to nearly 40%), so most of these distributions overlap. However, because correlations in the uncertainties are taken into account for relative EAR, the widths of these distributions decrease to less than 0.1% of the mean. These values are so small because the dose–response is linear and therefore the input uncertainties cancel very effectively when relative EAR is calculated. Table 9 shows that IMPT or 3DCRT produce the lowest total EAR estimates for all virtual patients with IMRT mostly producing the highest risk values as was the case for the FULL model. The absolute EAR values are larger for the linear model than the FULL model as the EAR continues to increase with dose and doesn’t plateau or decrease as can be the case in the FULL model.

**Table 9.** Linear model: absolute and relative EAR, summed over all organs for all five virtual patients

	EAR ($10,000PY^{-1}$)								EAR Relative to 3DCRT							
	IMPT		3DCRT		IMRT		VMAT		IMPT		3DCRT		IMRT		VMAT	
	Mean	CV	Mean	CV	Mean	CV	Mean	CV	Mean	CV	Mean	CV	Mean	CV	Mean	CV
Virtual Patient 1	89.5	14%	73.8	14%	108	16%	103	15%	1.22	0.1%	1	0.0%	1.48	0.1%	1.4	0.1%
Virtual Patient 2	123	20%	118	20%	146	21%	143	21%	1.04	0.0%	1	0.0%	1.24	0.0%	1.22	0.0%
Virtual Patient 3	160	31%	162	32%	227	34%	189	32%	0.99	0.0%	1	0.0%	1.41	0.1%	1.17	0.0%
Virtual Patient 4	196	31%	206	33%	267	36%	206	32%	0.95	0.0%	1	0.0%	1.3	0.0%	1	0.0%
Virtual Patient 5	230	33%	274	36%	315	38%	253	35%	0.84	0.0%	1	0.0%	1.15	0.0%	0.923	0.0%

CV, coefficient of variation; 3DCRT, three-dimensional conformal radiotherapy; EAR, excess absolute risk; IMPT, intensity modulated proton therapy; IMRT, intensity modulated radiotherapy; PY, per person per year;VMAT, volumetric modulated arc therapy.

CV is the ratio of the standard deviation to the mean.

Table 10 shows the significance of separation in the relative EAR distributions. For virtual patients 2 to 5 IMPT is significantly (*p <* 0.001 or three sigma) resolvable from both VMAT and IMRT. IMPT and 3DCRT have less than three sigma separating the relative EAR distributions for all. IMRT and VMAT overlap for virtual patients 1, 2 and 3 but are resolvable for the other two virtual patients.

**Table 10.** Linear model: significance of overlap of the relative EAR distributions for all virtual patients and modalities

	IMPT *vs* 3DCRT	IMPT *vs* IMRT	IMPT *vs* VMAT	3DCRT *vs* IMRT	3DCRT *vs* VMAT	IMRT *vs* VMAT
Virtual Patient 1	^*a*^	0.01	0.03	^*a*^	^*a*^	0.26
Virtual Patient 2	0.04	^*a*^	^*a*^	^*a*^	^*a*^	0.37
Virtual Patient 3	0.21	^*a*^	^*a*^	^*a*^	^*a*^	0.01
Virtual Patient 4	^*a*^	^*a*^	^*a*^	^*a*^	0.5	^*a*^
Virtual Patient 5	^*a*^	^*a*^	^*a*^	^*a*^	^*a*^	^*a*^

3DCRT, three-dimensionalconformal radiotherapy; EAR, excess absolute risk; IMPT, intensity modulated proton therapy; IMRT, intensity modulated radiotherapy; VMAT, volumetric modulated arc therapy.

a Means significant (*<*0.001), *i.e*. more than three standard deviations separating the distributions.

When comparing IMPT *vs* IMRT, for all virtual patients, the mean EAR difference is 52.9 per 10 000 person years. In terms of relative risk, the IMRT plans come with a 33% increase in risk relative to IMPT. For IMPT *vs* VMAT for all virtual patients, the mean EAR difference is 19.1 per 10 000 person years. In terms of relative risk, the VMAT plans come with a 12% increase in risk relative to IMPT. For IMPT *vs* 3DCRT for all virtual patients, the mean EAR difference is 7.1 per 10 000 person years. In terms of relative risk, the 3DCRT plans come with a 4% increase in risk relative to IMPT.

### EAR *vs* integral dose

**Figure 6.**
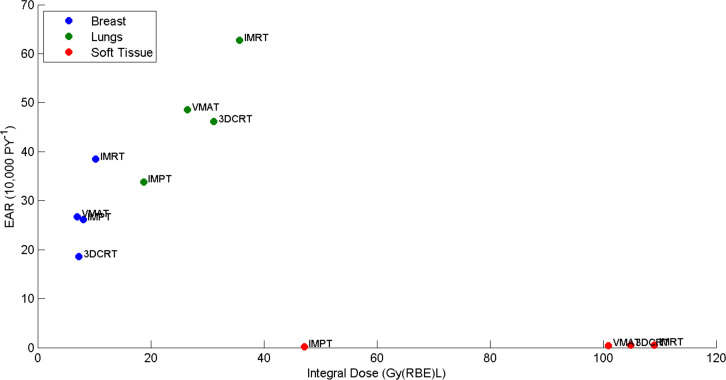
A plot showing the mean organ EAR per 10 000 person years *vs* organ integral dose for breasts, lungs and soft tissue for virtual patient 5. 3DCRT, three-dimensional conformal radiotherapy; EAR, excess absolute risk; IMPT, intensity modulated proton therapy; IMRT, intensity modulated radiotherapy; RBE, relative biological effectiveness; VMAT, volumetric modulatedarc therapy

Figure 6 shows the organ EAR estimates per 10,000 person years *vs* organ integral dose for breasts, lungs and soft tissue for virtual patient 5. Integral dose only gives an approximation to risk for an individual organ. This is emphasised by comparing VMAT with IMPT where the integral dose for the breasts is approximately the same, but the risk for VMAT is higher. This is because the dose response is not linear, and therefore risk is not directly proportional to integral dose but based on the 3D dose distribution.

Figure 6 also demonstrates how differences in organ susceptibility to second solid cancer can vary greatly. EAR *vs* integral dose for breasts and lungs (organs which have a high risk of second cancer) are shown, alongside the sarcoma induction EAR of the surrounding soft tissues. Even though the largest differences in integral dose between modalities occurs in the soft tissues the largest absolute differences in risk are seen in other organs at risk, *e.g.* lung and breast. Therefore, the risk is dominated by certain organs which, in the case of HL are in or close to the target region. Where modalities do differ largely in integral dose, *e.g*. bones and soft tissue, the absolute differences in EAR estimates are small.

**Figure 7.**
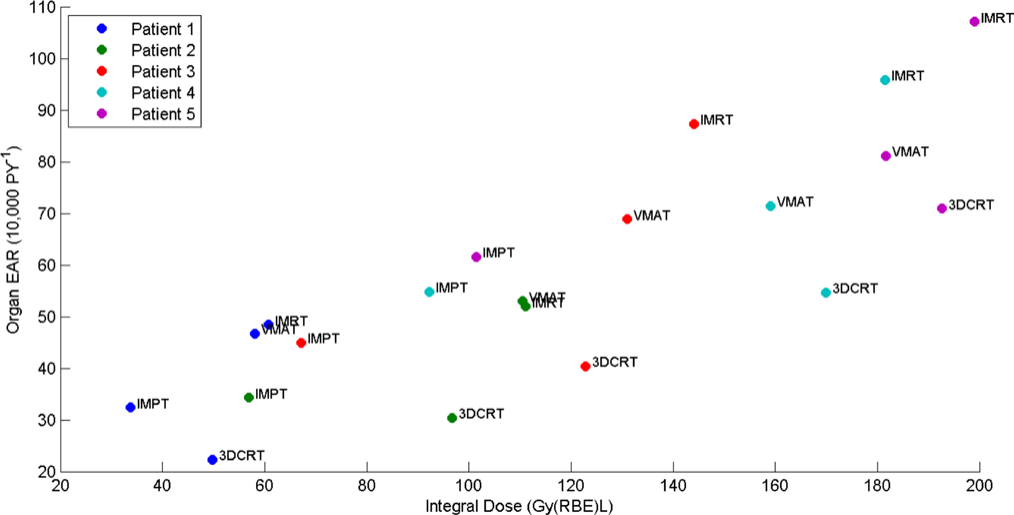
A plot showing mean EAR per 10,000 person years summed over all organs *vs* total integral dose for all virtual patients. 3DCRT, three-dimensional conformal radiotherapy; EAR, excess absolute risk; IMPT, intensity modulated proton therapy; IMRT, intensity modulated radiotherapy; RBE, relative biological effectiveness; VMAT, volumetric modulatedarc therapy.

Figure 7 shows the total EAR (summed over all organs) *vs* integral dose for all virtual patients. As the total EAR is dominated by the risk from the most susceptible organs, and not by the sarcoma risk in soft tissue or bone, the integral dose is not a good predictor for risk of a patient to obtain a second cancer. For all these virtual patients, 3DCRT has a much larger integral dose than IMPT and yet the total EAR is smaller. In summary, integral dose should only be considered directly proportional to total EAR if all the irradiated tissues had the same susceptibility to second cancer and the dose–response was linear.

## Discussion

On an organ-by-organ basis, relative EAR is a useful metric for distinguishing between two plans in terms of second cancer risk, as in most cases, each of the plans can be significantly resolved from the other three. For most organs, in most of the virtual patients, the modern photon plans (IMRT and VMAT) lead to higher EAR estimates than 3DCRT and IMPT. Virtual patient 5 has a larger PTV than virtual patient 1 and therefore higher EAR estimates to all organs, for all plans. The dominant organs by far, in terms of EAR, are lungs and breast. It is these organs which drive the total EAR estimates and therefore sparing dose in these organs can dramatically affect risk.

To put the organ EAR estimates in perspective, the per organ EARs for virtual patient 5 have been compared with the background incidences of these cancers per year at age 70 in Supplementary Table 7. For most organs, for all three photon plans, the estimated EAR is higher than the background incidence. In the case of the lungs, the IMRT plan increases the patient’s cancer risk to 3.7 times background risk. The IMPT plan is much better in terms of EAR for the stomach and liver, the EAR only increases the patient’s risk to 1.13 and 1.08 of background risk respectively. In short, these radiotherapy-induced risks are appreciable, should be modelled accurately and reduced where possible.

Supplementary Table 7.

The total EARs have parameter-driven uncertainties of about 13%, meaning that most of these distributions overlap. However, the width of the relative EAR distributions is dramatically reduced to between 2 and 5% of mean (similar to what was found for OED comparisons in Nguyen et al^14^). IMPT is associated with a statistically lower risk than VMAT or IMRT for all virtual patients. Relative EAR is therefore considered a useful metric in separating IMPT plans from the modern photon plans in terms of risk.

From summarising the results across all virtual patients, it is found that for IMPT *vs* IMRT the mean EAR difference is 34.3 per 10,000 person years, hence the IMRT plans come with a 67% increase in risk relative to IMPT. For IMPT *vs* VMAT, the mean EAR difference is 20.0 per 10,000 person years which equates to a 39% increase in risk relative to IMPT. IMPT and 3DCRT have been found to not be significantly resolvable for most virtual patients, hence the risk of radiotherapy-induced second cancer should be considered equivalent. In summary, IMPT leads to significantly decreased EAR with respect to modern photon plans (IMRT and VMAT). Similar findings can be found in Cella et al^15^ and Schneider et al^16^ where advanced RT techniques were shown to successfully spare organs at risk and to reduce the risk of radio-induced toxicities in HL patients, but with an increased risk of second malignant neoplasms.

It has been shown quantitatively in this analysis that risk is not proportional to integral dose. This is true for both individual organs (due to the non-linearity in the dose curve) and the total risk (where the bigger contributor is the large variation in organ sensitivity). This indicates the need to model risk in the manner presented in this paper, rather than simply using dose as a proxy for risk.

A linear model has also been employed to see how sensitive these results are to the shape of the dose-response curve. From comparing IMPT *vs* IMRT for all virtual patients, using linear model for each organ, the mean EAR difference is 52.9 per 10 000 person years. In terms of relative risk, the IMRT plans come with a 33% increase in risk relative to IMPT. This finding exemplifies the robustness of these results, which predict IMPT will cause a lower EAR than IMRT and VMAT even when the relationship of EAR to dose is dramatically altered.

Some limitations to our study are now discussed:

The results presented are plan-specific and not necessarily indicative of the modality as a whole. The possibility that the delivered dose may vary from the planned dose has not been taken into account. The IMPT plan used in this paper is actively scanned and therefore associated with low neutron dose.^17^ However, with large uncertainties in the biological effect of neutrons at different energies it may still be necessary to take neutron induced EAR into account in future work.

The spleen model was considered too uncertain due to a lack of clinical data in the organ to fit to. This is not expected to effect the results presented in an appreciable way as the spleen is mostly out of field for all RT plans.

An RBE of 1.1 has been assumed for every voxel in treatment volume and it is the RBE weighted dose that is D in the RED equations. This is an approximation. The National Commission of radiation protection report number 104 cites 2 values for the RBE of protons for an aberration endpoint, from Takatsuji et al^18^ and the work of Edwards et al^19^. The former found a Q value of 5.7 from proton energies of 4.9 MeV. However, the latter studied protons of 8.7 MeV from which the NCRP quote a Q value of 1.0. This implies that there is a strong energy dependence on the quality factor. Considering the treatment plans, the main contribution to the normal tissue integral dose will come from the plateau region of the Bragg curve. This portion of the Bragg curve consists predominantly of dose deposited by higher energy protons and will therefore have a quality factor of 1.0. In contrast, in the normal tissue distal to the target volume, although the quality factor maybe higher, the irradiated volume will be very much smaller. Therefore, it is safe to assume that the vast majority of normal tissues will be irradiated by protons with a quality factor much closer to 1.0 than the 5.7 found for very low energies; 1.1 has been used in this study.

The same ABVD chemotherapy regime would be administered to all virtual patients, regardless of radiotherapy modality. The leukaemia risk estimate is outside the scope of this paper as it is assumed to be caused mainly by chemotherapy, rather than the radiotherapy. This means that synergistic effects between chemotherapy and radiotherapy are not considered.

## Conclusion

Relative EAR is a useful metric for distinguishing between two treatment plans in terms of second cancer risk, on an organ-by-organ basis and for the patient as a whole. Parameter-driven uncertainties on total EAR estimations are around 13%, this decreases to around 2–5% for relative EAR modality comparisons. IMPT leads to a significantly decreased EAR with respect to modern photon plans (IMRT and VMAT). Using protons rather than IMRT could decrease these virtual patients’ risk of cancer by more than 40%. A comparison using a linear model exemplifies the robustness of these results, which predict IMPT will cause a lower EAR than IMRT, even when the relationship of EAR to dose is dramatically altered. It has also been shown quantitatively in this analysis that risk is not proportional to integral dose. This is true for both individual organs (due to the non-linearity in the dose curve) and the total risk (where the bigger contributor is the large variation in organ sensitivity).
