# Supplementary material for: Comparing second cancer risk for multiple radiotherapy modalities in survivors of hodgkin lymphoma
Source: Br J Radiol. 2021 Apr 9;94(1121):20200354. doi: 10.1259/bjr.20200354 (PMC8506169; doi:10.1259/bjr.20200354)
Supplement: Supplementary Table 1. [file bjr.20200354.suppl-01.docx]

Table 1 A dosimetric summary of organs for the four treatment plans for virtual patient 1 detailing minimum, maximum, mean and integral doses within the volume enclosed by each contoured structure. Proton dose: D_RBE_ Gy(RBE) = RBE ×D(Gy) where RBE (Relative Biological Effectiveness) is assumed to be 1.1 in every voxel. The integral dose is given in units of GyLitres which has been abbreviated to GyL.

| Structure |  | IMPT Dose [Gy(RBE)] | | | | 3DCRT Dose (Gy) | | | | IMRT Dose (Gy) | | | | VMAT Dose (Gy) | | | |
| --- | --- | --- | --- | --- | --- | --- | --- | --- | --- | --- | --- | --- | --- | --- | --- | --- | --- |
|  | Volume | Min | Max | mean | Integral | Min | Max | mean | Integral | Min | Max | mean | Integral | Min | Max | mean | Integral |
|  | cm${}^{3}$ |  |  |  | Gy(RBE)L |  |  |  | GyL |  |  |  | GyL |  |  |  | GyL |
| Breast | 901 | 0.0 | 14.6 | 2.6 | 2.32 | 0.0 | 32.6 | 0.7 | 0.67 | 0.0 | 15.8 | 2.8 | 2.52 | 0.0 | 20.0 | 2.5 | 2.28 |
| Oesophagus | 28 | 0.0 | 33.1 | 9.2 | 0.25 | 0.2 | 30.9 | 9.2 | 0.25 | 0.1 | 32.3 | 8.8 | 0.24 | 0.2 | 31.6 | 8.8 | 0.24 |
| Heart | 610 | 0.0 | 32.1 | 2.3 | 1.39 | 0.2 | 33.2 | 5.4 | 3.32 | 0.1 | 31.8 | 2.7 | 1.67 | 0.1 | 31.6 | 3.3 | 2.00 |
| Liver | 1912 | 0.0 | 0.0 | 0.0 | 0.0 | 0.0 | 0.4 | 0.1 | 0.21 | 0.0 | 0.4 | 0.1 | 0.11 | 0.0 | 0.5 | 0.1 | 0.2 |
| Lungs | 2398 | 0.0 | 33.2 | 4.5 | 10.9 | 0.1 | 33.8 | 4.3 | 10.37 | 0.1 | 32.1 | 6.7 | 16.1 | 0.1 | 31.3 | 6.2 | 14.85 |
| Pharynx | 37 | 0.0 | 0.0 | 0.0 | 0.0 | 0.1 | 0.4 | 0.2 | 0.01 | 0.0 | 0.2 | 0.1 | 0.0 | 0.1 | 0.4 | 0.2 | 0.01 |
| Spinal Cord | 79 | 0.0 | 15.2 | 0.6 | 0.05 | 0.0 | 31.2 | 4 | 0.31 | 0.0 | 22.7 | 2.2 | 0.18 | 0.0 | 23.7 | 2.4 | 0.19 |
| Spleen | 353 | 0.0 | 0.0 | 0.0 | 0.0 | 0.0 | 0.2 | 0.1 | 0.03 | 0.0 | 0.2 | 0.0 | 0.01 | 0.0 | 0.3 | 0.1 | 0.03 |
| Stomach | 713 | 0.0 | 0.0 | 0.0 | 0.0 | 0.0 | 0.4 | 0.1 | 0.09 | 0.0 | 0.4 | 0.1 | 0.05 | 0.0 | 0.4 | 0.1 | 0.07 |
| Thyroid | 14 | 0.0 | 0.0 | 0.0 | 0.0 | 0.3 | 0.8 | 0.5 | 0.01 | 0.2 | 0.6 | 0.3 | 0.0 | 0.3 | 1.1 | 0.6 | 0.01 |
| Vessels | 268 | 0.0 | 33 | 23.1 | 6.2 | 0.2 | 33.1 | 23.4 | 6.29 | 0.1 | 32.6 | 22.5 | 6.04 | 0.2 | 32 | 22.5 | 6.04 |
| Bone | 2478 | 0.0 | 32.4 | 1.3 | 3.32 | 0.0 | 33.9 | 3.1 | 7.74 | 0.0 | 32.2 | 2.8 | 6.93 | 0.0 | 31.8 | 2.8 | 6.94 |
| Soft Tissue | 16543 | 0.0 | 33.1 | 0.6 | 9.32 | 0.0 | 33.9 | 1.2 | 20.55 | 0.0 | 32.4 | 1.6 | 26.87 | 0.0 | 32.1 | 1.5 | 25.29 |
