# Supplementary material for: Comparing second cancer risk for multiple radiotherapy modalities in survivors of hodgkin lymphoma
Source: Br J Radiol. 2021 Apr 9;94(1121):20200354. doi: 10.1259/bjr.20200354 (PMC8506169; doi:10.1259/bjr.20200354)
Supplement: Supplementary Table 2. [file bjr.20200354.suppl-02.docx]

Table 2 A dosimetric summary of target structures for the four treatment plans for virtual patient 1 detailing 5*^th^* percentile, maximum, mean and integral doses within the volume enclosed by each contoured structure. Proton dose: *D_RBE_ Gy*(*RBE*) = *RBE* ×*D*(*Gy*) where RBE (Relative Biological Effectiveness) is assumed to be 1.1 in every voxel. The integral dose is given in units of GyLitres which has been abbreviated to GyL.

| Structure |  | IMPT Dose [Gy(RBE)] | | | | 3DCRT Dose (Gy) | | | | IMRT Dose (Gy) | | | | VMAT Dose (Gy) | | | |
| --- | --- | --- | --- | --- | --- | --- | --- | --- | --- | --- | --- | --- | --- | --- | --- | --- | --- |
|  | Vol | 5^th^ | Max | Mean | Integral | 5^th^ | Max | Mean | Integral | 5^th^ | Max | Mean | Integral | 5^th^ | Max | Mean | Integral |
|  | cm${}^{3}$ |  |  |  | Gy(RBE)L |  |  |  | GyL |  |  |  | GyL |  |  |  | GyL |
| CTV1 Upper med | 145 | 30.5 | 33.1 | 31.2 | 4.52 | 30.1 | 32.8 | 30.9 | 4.48 | 31.5 | 32.6 | 31.9 | 4.61 | 30.6 | 31.9 | 31.1 | 4.49 |
| PTV1 Upper Med | 112 | 29.6 | 33.2 | 30.9 | 3.46 | 29.9 | 33.1 | 30.9 | 3.47 | 29.6 | 32.3 | 31.1 | 3.47 | 29.5 | 32.1 | 30.9 | 3.45 |
