# Supplementary material for: Comparing second cancer risk for multiple radiotherapy modalities in survivors of hodgkin lymphoma
Source: Br J Radiol. 2021 Apr 9;94(1121):20200354. doi: 10.1259/bjr.20200354 (PMC8506169; doi:10.1259/bjr.20200354)
Supplement: Supplementary Table 3. [file bjr.20200354.suppl-03.docx]

##

Table 3 A dosimetric summary of the four treatment plans for virtual patient 5 detailing minimum, maximum, mean and integral doses within the volume enclosed by each contoured structure. Proton dose: *D_RBE_ Gy*(*RBE*) = *RBE*×*D*(*Gy*) where RBE (Relative Biological Effectiveness) is assumed to be 1.1 in every voxel. The integral dose is given in units of GyLitres which has been abbreviated to GyL.

| Structure |  | IMPT Dose [Gy(RBE)] | | | | 3DCRT Dose (Gy) | | | | IMRT Dose (Gy) | | | | VMAT Dose (Gy) | | | |
| --- | --- | --- | --- | --- | --- | --- | --- | --- | --- | --- | --- | --- | --- | --- | --- | --- | --- |
|  | Volume | Min | Max | Mean | Integral | Min | Max | Mean | Integral | Min | Max | Mean | Integral | Min | Max | Mean | Integral |
|  | cm${}^{3}$ |  |  |  | Gy(RBE)L |  |  |  | GyL |  |  |  | GyL |  |  |  | GyL |
| Breast | 901 | 0.0 | 33 | 8.9 | 8.02 | 0.0 | 33.2 | 8.1 | 7.27 | 0.5 | 32.7 | 11.4 | 10.25 | 0.0 | 35.4 | 7.7 | 6.96 |
| Oesophagus | 28 | 0.0 | 33.4 | 22.1 | 0.61 | 1.2 | 32.1 | 22.2 | 0.61 | 1.2 | 33.1 | 21.8 | 0.60 | 1.4 | 37.7 | 22.2 | 0.61 |
| Heart | 610 | 0.0 | 33.8 | 14.5 | 8.84 | 1.3 | 32.7 | 23.6 | 14.39 | 1.1 | 32.3 | 18.1 | 11.02 | 1.1 | 36.1 | 22.3 | 13.60 |
| Liver | 1912 | 0.0 | 8.0 | 0.1 | 0.10 | 0.0 | 5.1 | 0.4 | 0.81 | 0.0 | 8.5 | 0.6 | 1.11 | 0.0 | 7.2 | 0.6 | 1.10 |
| Lungs | 2398 | 0.0 | 34.4 | 7.8 | 18.71 | 0.2 | 32.8 | 13 | 31.13 | 0.6 | 32.4 | 14.9 | 35.74 | 0.5 | 36.7 | 11.0 | 26.46 |
| Pharynx | 37 | 0.0 | 33.6 | 2.7 | 0.10 | 0.7 | 32.0 | 3.9 | 0.15 | 0.6 | 33.0 | 3.6 | 0.13 | 0.6 | 32.7 | 3.6 | 0.13 |
| Spinal Cord | 79 | 0.0 | 24.5 | 2.3 | 0.19 | 0.0 | 31.8 | 10.1 | 0.80 | 0.0 | 32.5 | 7.8 | 0.62 | 0.0 | 35.7 | 9.5 | 0.75 |
| Spleen | 353 | 0.0 | 0.0 | 0.0 | 0.00 | 0.2 | 2.0 | 0.6 | 0.22 | 0.1 | 2.2 | 0.5 | 0.16 | 0.1 | 1.7 | 0.5 | 0.17 |
| Stomach | 713 | 0.0 | 9.4 | 0.1 | 0.04 | 0.1 | 22.2 | 1.2 | 0.88 | 0.1 | 11.5 | 1.0 | 0.72 | 0.0 | 6.2 | 0.8 | 0.54 |
| Thyroid | 14 | 28.4 | 34.2 | 32.3 | 0.46 | 15.4 | 32.2 | 30.9 | 0.44 | 18.7 | 33.5 | 31.4 | 0.45 | 19.5 | 37.2 | 31.9 | 0.46 |
| Vessels | 268 | 0.0 | 33.9 | 29.2 | 7.84 | 1.1 | 32.9 | 29.9 | 8.04 | 1.1 | 33.3 | 29.4 | 7.9 | 1.3 | 37.2 | 30.4 | 8.15 |
| Bone | 2478 | 0.0 | 33.7 | 3.8 | 9.44 | 0.0 | 33.3 | 9.3 | 22.99 | 0.0 | 33.5 | 8.6 | 21.33 | 0.0 | 37.9 | 8.8 | 21.75 |
| Soft Tissue | 16543 | 0.0 | 34.5 | 2.9 | 47.15 | 0.0 | 33.5 | 6.3 | 104.91 | 0.0 | 33.8 | 6.6 | 109.05 | 0.0 | 37.8 | 6.1 | 100.93 |
