# Supplementary material for: Comparing second cancer risk for multiple radiotherapy modalities in survivors of hodgkin lymphoma
Source: Br J Radiol. 2021 Apr 9;94(1121):20200354. doi: 10.1259/bjr.20200354 (PMC8506169; doi:10.1259/bjr.20200354)
Supplement: Supplementary Table 4. [file bjr.20200354.suppl-04.docx]

Table 4 A dosimetric summary of target structures for the four treatment plans for virtual patient 5 detailing 5*^th^* percentile, maximum, mean and integral doses within the volume enclosed by each contoured structure. Proton dose: D_RBE_ Gy(RBE) = RBE ×D(Gy) where RBE (Relative Biological Effectiveness) is assumed to be 1.1 in every voxel. The integral dose is given in units of GyLitres which has been abbreviated to GyL

| Structure |  | IMPT Dose [Gy(RBE)] | | | | 3DCRT Dose (Gy) | | | | IMRT Dose (Gy) | | | | VMAT Dose (Gy) | | | |
| --- | --- | --- | --- | --- | --- | --- | --- | --- | --- | --- | --- | --- | --- | --- | --- | --- | --- |
|  | Vol | 5^th^ | Max | Mean | Integral | 5^th^ | Max | Mean | Integral | 5^th^ | Max | Mean | Integral | 5^th^ | Max | Mean | Integral |
|  | cm${}^{3}$ |  |  |  | Gy(RBE)L |  |  |  | GyL |  |  |  | GyL |  |  |  | GyL |
| CTV1 Upper med | 145 | 31.0 | 33.9 | 31.5 | 4.56 | 30.5 | 32.7 | 31.2 | 4.51 | 31.1 | 32.3 | 31.4 | 4.55 | 31.2 | 34.5 | 32.3 | 4.67 |
| CTV2 Supraclav | 166 | 31.6 | 34.1 | 32.2 | 5.34 | 31.0 | 33.2 | 31.6 | 5.25 | 31.5 | 33.8 | 32.3 | 5.36 | 31.2 | 37.9 | 32.6 | 5.42 |
| CTV3 Lower Med | 39 | 30.8 | 33.0 | 31.4 | 1.23 | 29.9 | 31.5 | 30.4 | 1.19 | 31.2 | 32.3 | 31.6 | 1.24 | 30.6 | 34.8 | 31.4 | 1.23 |
| CTV4 axilla | 112 | 31.2 | 33.4 | 31.6 | 3.56 | 30.4 | 32.4 | 31.1 | 3.49 | 31.6 | 33.1 | 32.3 | 3.63 | 31.5 | 35.8 | 32.5 | 3.66 |
| CTV5 Epicardial | 40 | 31.3 | 34.3 | 31.8 | 1.27 | 30.7 | 33.0 | 31.6 | 1.26 | 31.1 | 32.4 | 31.7 | 1.27 | 30.1 | 36.8 | 32.1 | 1.28 |
| PTV1 Upper Med | 112 | 29.9 | 33.8 | 31.0 | 3.48 | 30.4 | 32.9 | 31.3 | 3.51 | 29.9 | 32.2 | 30.7 | 3.45 | 30.3 | 35.1 | 31.8 | 3.56 |
| PTV2 Supraclav | 172 | 30.5 | 34.3 | 31.7 | 5.43 | 30.4 | 33.2 | 31.2 | 5.36 | 30.6 | 33.6 | 31.6 | 5.42 | 30.9 | 37.7 | 32.3 | 5.55 |
| PTV3 Lower med | 42 | 29.4 | 32.9 | 30.7 | 1.29 | 29.4 | 31.7 | 30.3 | 1.28 | 29.4 | 31.9 | 30.5 | 1.28 | 30.0 | 35.3 | 31.4 | 1.32 |
| PTV4 axilla | 112 | 29.8 | 33.3 | 31.0 | 3.48 | 29.5 | 32.5 | 30.5 | 3.43 | 30.6 | 33 | 31.4 | 3.53 | 30.1 | 35.4 | 31.5 | 3.54 |
| PTV5 Epicardial | 66 | 29.3 | 34.4 | 31.0 | 2.05 | 30.4 | 33.1 | 31.5 | 2.08 | 29.8 | 32.3 | 30.9 | 2.04 | 27.5 | 36.9 | 30.4 | 2.01 |
