# Supplementary material for: Comparing second cancer risk for multiple radiotherapy modalities in survivors of hodgkin lymphoma
Source: Br J Radiol. 2021 Apr 9;94(1121):20200354. doi: 10.1259/bjr.20200354 (PMC8506169; doi:10.1259/bjr.20200354)
Supplement: Supplementary Table 5. [file bjr.20200354.suppl-05.docx]

Table 5 Significance of overlap of the relative EAR distributions for all organs and modalities for virtual patient 1. “∗” means significant (<0.001), i.e. more than 3 sigma separating the distributions. “-” indicates this organ is excluded either because this structure is out of field or there is considered no risk of second solid cancer (e.g. heart).

| Structure | IMPT vs 3DCRT | IMPT vs IMRT | IMPT vs VMAT | 3DCRT vs IMRT | 3DCRT vs VMAT | IMRT vs VMAT |
| --- | --- | --- | --- | --- | --- | --- |
| Breast | $*$ | $*$ | $*$ | $*$ | $*$ | 0.01 |
| Oesophagus | $*$ | $*$ | $*$ | $*$ | 0.07 | $*$ |
| Heart | - | - | - | - | - | - |
| Liver | $*$ | $*$ | $*$ | $*$ | $*$ | $*$ |
| Lungs | $*$ | $*$ | $*$ | $*$ | $*$ | 0.01 |
| Pharynx | - | - | - | - | - | - |
| Spinal Cord | $*$ | $*$ | $*$ | $*$ | $*$ | 0.26 |
| Spleen | - | - | - | - | - | - |
| Stomach | $*$ | $*$ | $*$ | $*$ | $*$ | $*$ |
| Thyroid | $*$ | $*$ | $*$ | $*$ | $*$ | $*$ |
| Vessels | - | - | - | - | - | - |
| Bone | $*$ | $*$ | $*$ | $*$ | $*$ | $*$ |
| Soft tissue | $*$ | $*$ | $*$ | $*$ | $*$ | $*$ |
