# Supplementary material for: Comparing second cancer risk for multiple radiotherapy modalities in survivors of hodgkin lymphoma
Source: Br J Radiol. 2021 Apr 9;94(1121):20200354. doi: 10.1259/bjr.20200354 (PMC8506169; doi:10.1259/bjr.20200354)
Supplement: Supplementary Table 7. [file bjr.20200354.suppl-07.docx]

## Background cancer incidence comparison

Table 7 Background incidences of female cancers at age 70 per 10,000 (Source:https://www.cancerresearchuk.org). Comparison with per organ EAR values for virtual patient 5.

| Organ | Background  (per 10,000) | $\frac{EAR}{Background}$ | | | |
| --- | --- | --- | --- | --- | --- |
|  |  | IMPT | 3DCRT | IMRT | VMAT |
| Breast | 39.1 | 69% | 50% | 102% | 71% |
| Oesophagus | 1.75 | 109% | 137% | 137% | 137% |
| Liver | 1.18 | 8% | 68% | 76% | 76% |
| Lung | 23.6 | 146% | 200% | 271% | 210% |
| Head and Neck | 3.02 | 40% | 66% | 60% | 63% |
| Stomach | 1.53 | 13% | 183% | 170% | 157% |
| Thyroid | 1.09 | 239% | 257% | 248% | 248% |
